# Gut Microbiota and Metabolome Description of Antibiotic-Treated Neonates From Parturients With Intrauterine Infection

**DOI:** 10.3389/fcimb.2022.817832

**Published:** 2022-03-18

**Authors:** Huitao Li, Lei Fu, Xueyu Chen, Heng Xu, Qinlong Jing, Chuanzhong Yang, Zhengwei Wan, Yiran Chen

**Affiliations:** ^1^ Affiliated Shenzhen Maternity & Child Healthcare Hospital, Southern Medical University, Shenzhen, China; ^2^ Institute of Public Health, Guangzhou Medical University & Guangzhou Center for Disease Control and Prevention, Guangzhou, China; ^3^ Department of Health Management Center & Institute of Health Management, Sichuan Provincial People’s Hospital, University of Electronic Science and Technology of China, Chengdu, China

**Keywords:** intrauterine infection, gut microbiota, metabolome, neonate, antibiotic

## Abstract

Intrauterine infection is linked to adverse pregnancy outcomes in pregnant women. Neonates from parturients with intrauterine infection are usually treated with antibiotics, but their gut microbiota and metabolome are seldom studied. In this study, we collected fecal samples from antibiotic-treated neonates of parturients with intrauterine infection (intrauterine infection group), parturients with non-intrauterine infection (antibiotic group), and untreated neonates of healthy parturients (control group). 16S rRNA gene sequencing and untargeted metabolomics analyses were performed. Our results revealed that the α-diversity of intrauterine infection group differed from that of control group. There were significant differences in β-diversity between intrauterine infection group and control group, between antibiotic group and the control group, but there was no difference between the intrauterine infection and antibiotic groups, implying that antibiotic use has an obvious effect on β-diversity and that the effects of intrauterine infection on β-diversity cannot be identified. *Enterococcus* was more abundant in intrauterine infection and antibiotic groups than in control group. Gut metabolite differences in intrauterine infection group and antibiotic group (only in negative ion mode) from control group were observed, but no difference between intrauterine infection group and antibiotic group was observed. N-formyl-L-methionine was the most discriminant metabolite between intrauterine infection group and control group. Primary and secondary bile acid biosynthesis, bile secretion, and cholesterol metabolism pathways were altered, and the abundances of bile acids and bile salts were altered in intrauterine infection group compared with control group. Alterations in cholesterol metabolism, arginine biosynthesis and bile secretion pathways were observed both in intrauterine infection and antibiotic groups, which might be caused by the use of antibiotics. In conclusion, we provided a preliminary description of the gut microbiota and gut metabolites in antibiotics-treated neonates from intrauterine infection parturients. Our findings did not show intrauterine infection has a separate role in neonatal gut microbiota dysbiosis, while supporting the idea that antibiotics should be used with caution during neonatal therapy.

## Introduction

Recently, the neonatal gut microbiota has attracted much attention (La Rosa et al., 2014; Stinson et al., 2017; Roubaud-Baudron et al., 2019). The diversity and abundance of gut bacteria in neonates are very low at birth, and this can easily be disturbed. Epidemiological studies have shown that perturbation of gut microbiota in early life has long-term effects, which may increase the risk of diseases such as obesity, immune dysfunction, and abnormalities in the nervous system later in life (Hsiao et al., 2013; Vangay et al., 2015; Vatanen et al., 2016), causing great harm and economic burden to society.

Neonatal health can be affected by many variables (Schei et al., 2017; Kazmi et al., 2019; Chen et al., 2021). Intrauterine infections have been linked to adverse pregnancy outcomes such as premature rupture of fetal membranes, preterm birth, and fetal infection (Goncalves et al., 2002; Romero et al., 2003). The prevalence, pathways, stages, and pathophysiology of ascending intrauterine infections have been studied. Understanding intestinal health according to gut microbiota and gut metabolic function of neonates of parturients with intrauterine infection is important, and only a few studies have been reported on this topic.

Antibiotics are generally administered to neonates of parturients with intrauterine infection immediately after birth. Although antibiotic treatment can prevent infectious diseases and improve survival rates, early life antibiotic exposure can affect the normal establishment of the intestinal microbiota. Many diseases are associated with antibiotics during the neonatal period (Carl et al., 2014; Warner et al., 2016; Leong et al., 2020). How intrauterine infection and antibiotic therapy modify the gut microbiota of newborns and impact gut microbial metabolism is an emerging subject.

In this study, we enrolled three groups of neonates, including neonates treated with antibiotics, who were delivered by parturients with intrauterine infection, neonates treated with antibiotics from parturients with non-intrauterine infection, and untreated neonates of healthy parturients. Sequencing and untargeted metabolomics were used to provide a basic description of the gut microbiota and microbial metabolism of antibiotic-treated neonates of parturients with intrauterine infection and focused on two points: 1) gut microbiota and gut microbial metabolism differences between neonates with early antibiotic administration born to parturients with intrauterine infection and non-intrauterine infection; 2) reshaping of gut microbiota and the change in gut microbial metabolism in antibiotic-treated neonates of parturients with intrauterine infection compared with healthy neonates.

## Materials and Methods

The parturients and neonates in this study were recruited from the Shenzhen Maternity and Child Healthcare Hospital (Shenzhen, Guangdong, China). This study was approved by the Medical Ethics Committee of the Shenzhen Maternal and Child Health Hospital (SFYLS [2021]006). All parturients signed informed consent for their neonates. Fecal samples and blood samples from all neonates were collected during the first week after birth. Metadata of parturients, such as age, pre-pregnancy BMI, and gestational age, were recorded. Metadata of neonates, such as gender, delivery mode, birth weight, feeding mode, antibiotics administration and type, bacterial cultures, and blood cell counts on the day of fecal sampling, were recorded. A total of 69 fecal samples of 69 neonates were collected. DNA from fecal samples was extracted, and 16S rRNA gene sequencing was conducted for PCR products that met the sequencing requirements. The raw reads were deposited into the European Nucleotide Archive database (accession number: PRJEB48156).

Untargeted metabolomics by LC-MS were conducted for all fecal samples. Details of participant information (**Table S1**), application of antibiotics, sample processing, DNA extraction and sequencing, untargeted metabolomics, and data analysis are shown in the **Supplementary Methods**.

## Results

### Study Participants

A total of 23 neonates treated with antibiotics from parturients with intrauterine infection (intrauterine infection group), 16 neonates treated with antibiotics born to parturients with non-intrauterine infection (antibiotic group), and 30 untreated neonates born to healthy parturients (control group) were included in this study. Sixty-nine fecal samples from all neonates were used for the untargeted metabolome and 60 PCR products (18 in the intrauterine infection group, 12 in the antibiotic group, and 30 in the control group) that met the sequencing requirements were used for 16S rRNA sequencing. Kruskal–Wallis tests were used to compare the age, pre-pregnancy BMI, gestational age, and birth weight among the three groups. Chi-squared tests were used to compare gender and delivery mode among the three groups. There were no differences in parturient age, pre-pregnancy BMI, gestational age, neonatal sex, delivery mode, birth weight, and feeding mode among the three groups (**Table 1**). The Mann–Whitney test showed no difference in antibiotic treatment time between the intrauterine infection and antibiotic groups (**Table 1**).

**Table 1:** Clinical information for all mothers and neonates.

Variables	Intrauterine infection group (n = 23)	Antibiotic group (n = 16)	Control group (n = 30)	P value
**Maternal**				
Age, years (mean ± SD)	30.96 ± 5.701	31.25 ± 5.310	31.6 ± 4.553	0.4929
Pre-pregnancy BMI, kg/m^2^ (mean ± SD)	20.75 ± 2.257	20.47 ± 1.801	21.19 ± 2.925	0.8787
Gestational age, weeks (mean ± SD)	39.37 ± 1.004	38.8 ± 1.389	38.56 ± 2.142	0.314
**Neonatal**				
Gender (%)				0.1608
Boy	10 (43.48%)	11 (68.75%)	20 (66.67%)	
Girl	13 (56.52%)	5 (31.25%)	10 (33.33%)	
Delivery mode				0.9681
Cesarean section	11 (47.83%)	7 (43.73%)	14 (46.67%)	
Vaginal	12 (52.17%)	9 (56.25%)	16 (53.33%)	
Birth weight, g (mean ± SD)	3304 ± 366.4	3128 ± 440.2	3144 ± 560.1	0.4975
Antibiotics usage (%)	100	100	0	
Antibiotics treatment time, day (mean ± SD)	4.778 ± 1.309	4.167 ± 1.193	0	0.1553
Feeding mode				
Mixed fed (formula + breast-feeding) (%)	100	100	100	

### Gut Microbiota Imbalance of Antibiotic-Treated Neonates of Parturients With Intrauterine Infection

**Figure 1:**
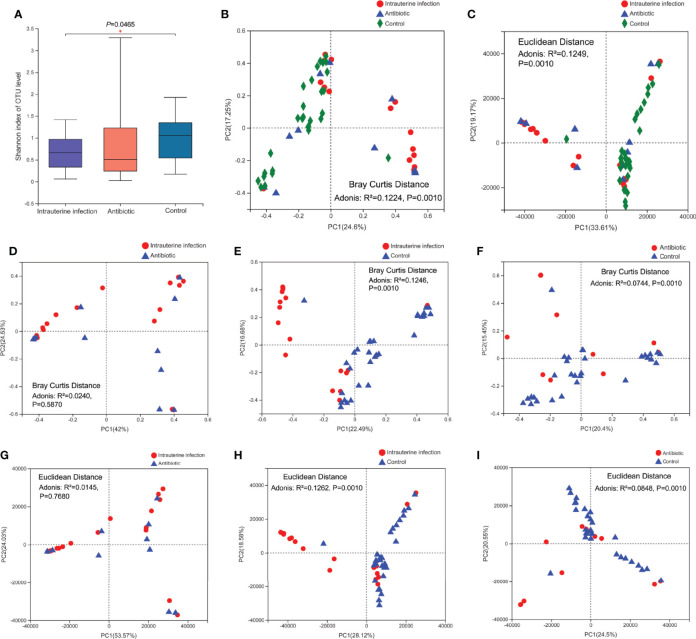
**(A)** Gut microbiota α-diversity of three groups. PCoA of gut microbiota of three groups based on the Bray–Curtis distance **(B)** and Euclidean distance **(C)**. PCoA of the gut microbiota based on the Bray–Curtis distance between the intrauterine infection group and antibiotic group **(D)**, between the intrauterine infection group and control group **(E)**, and between the antibiotic group and control group **(F)**. PCoA of the gut microbiota based on the Euclidean distance between the intrauterine infection group and antibiotic group **(G)**, between the intrauterine infection group and control group **(H)**, and between the antibiotic group and control group **(I)**. “*” means P value smaller than 0.05.

Compared with the healthy control group, a lower Shannon index was observed in the intrauterine infection group (*P* = 0.0465, **Figure 1A**), while PD_whole_tree and observed_OTUs were similar (data not shown). The Shannon index, PD_whole_tree, and observed_OTUs were not different between the intrauterine infection and antibiotic groups, as well as between the antibiotic and control groups (data not shown).

The gut microbiota β-diversity of the three groups was then compared. Adonis analyses based on Bray–Curtis and Euclidean distances showed that the β-diversity among the three groups was significantly different (*P* = 0.0010 for both) (**Figures 1B, C**). Adonis analyses based on Bray–Curtis and Euclidean distances revealed significant differences in β-diversity between the intrauterine infection group and control group and between the antibiotic and control groups (*P* = 0.0010 for all, **Figures 1E, F, H, I**), suggesting an obvious effect of antibiotic use on β-diversity in the first week after birth. The β-diversity of gut microbiota between the intrauterine infection and antibiotic groups was not significantly different (**Figures 1D, G**), which might suggest the effects of intrauterine infection on β-diversity cannot be identified.

**Figure 2:**
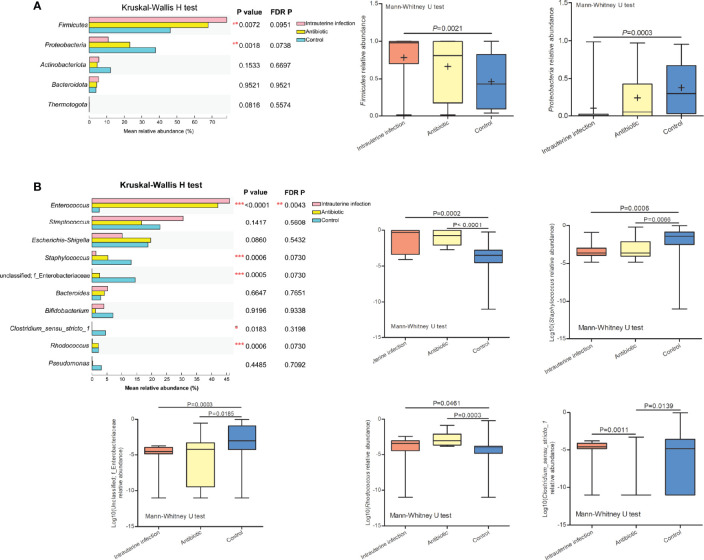
**(A)** Relative abundance of the top five phyla among the three groups, and the significantly different phyla among the three groups. **(B)** Relative abundance of the top ten genera among the three groups, and the log_10_ relative abundance of significantly different genera among the three groups. “*” means P or FDR-adjusted P values smaller than 0.05; “**” means P or FDR-adjusted P values smaller than 0.01; “***” means P or FDR-adjusted P values smaller than 0.001.

At the phylum level, significant differences of *Firmicutes* and *Proteobacteria* relative abundances among three groups were detected (**Figure 2A**). *Firmicutes* was more abundant in the intrauterine infection group than in the control group, and *Proteobacteria* was less abundant in the intrauterine infection group than in the control group. At the genus level, the relative abundances of *Enterococcus*, *Staphylococcus*, unclassified Enterobacteriaceae, *Clostridium_sensu_stricto_1*, and *Rhodococcus* were different among the three groups (**Figure 2B**). We found that the mean relative abundance of *Enterococcus* showed the most remarkable discrepancy between the intrauterine infection and control groups (46.08% vs. 2.45%). *Enterococcus* relative abundances were different between the antibiotic and control groups, and were not different between the intrauterine infection and antibiotic groups. Relative abundances of *Staphylococcus*, unclassified Enterobacteriaceae and *Rhodococcus* also showed differences between intrauterine infection group and control group, and between antibiotic group and control group, while no difference were detected between intrauterine infection group and antibiotic group. Relative abundance of *Clostridium_sensu_stricto_1* was different between antibiotic group and control group. These results may suggest the effect of antibiotics on relative abundance of bacteria in intrauterine infection group. We further detected the relationship between antibiotic treatment time and relative abundance of the top ten genera, and no correlation was found (data not shown).

### *Enterococcus* Was Associated With Neonatal Infection and Inflammation

**Figure 3:**
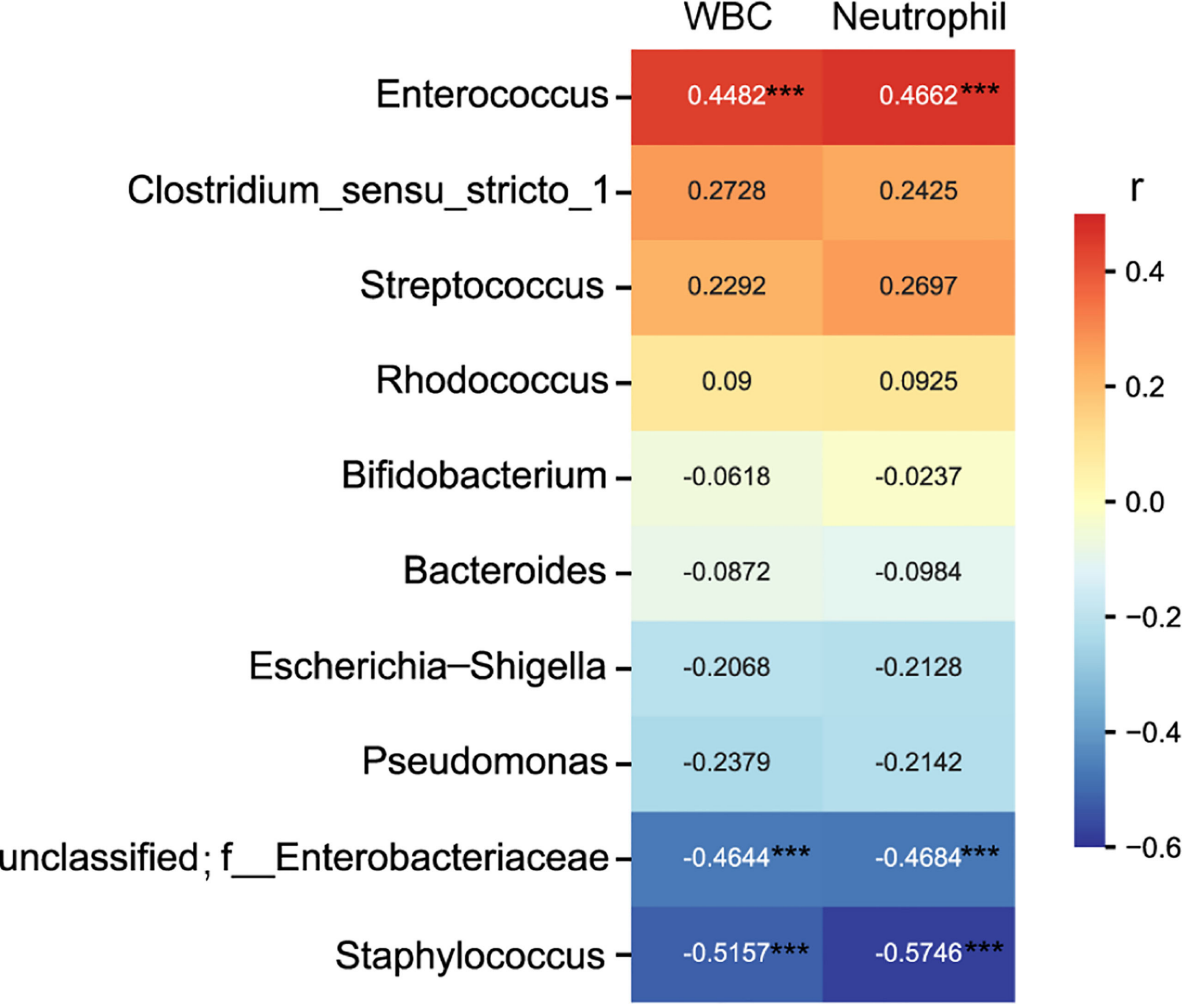
Heatmap of the correlation coefficients between the top ten genera and neonatal white blood cell and neutrophil counts. Spearman’s correlation coefficients are given in the heatmap, and significant correlations are represented by ‘***’ with FDR corrected *P* < 0.001.

Spearman rank correlation test suggested that neonatal gut microbiota correlated with neonatal infection and inflammation (n = 60 samples). Among the top ten genera, *Enterococcus* was positively associated with neonate neutrophil and WBC counts, and *Staphylococcus* and unclassified Enterobacteriaceae were negatively associated with neonate neutrophil and WBC counts (FDR P < 0.001 for all) (**Figure 3**), indicating that they were related to infection and inflammation. *Clostridium_sensu_stricto_1, Streptococcus, Rhodococcus, Bifidobacterium, Bacteroides, Escherichia-Shigella*, and *Pseudomonas* were not associated with neonate neutrophil and WBC counts (data not shown).

### Alterations of Gut Metabolome and Metabolic Pathways in Antibiotics-Treated Neonates of Parturients With Intrauterine Infection

**Figure 4:**
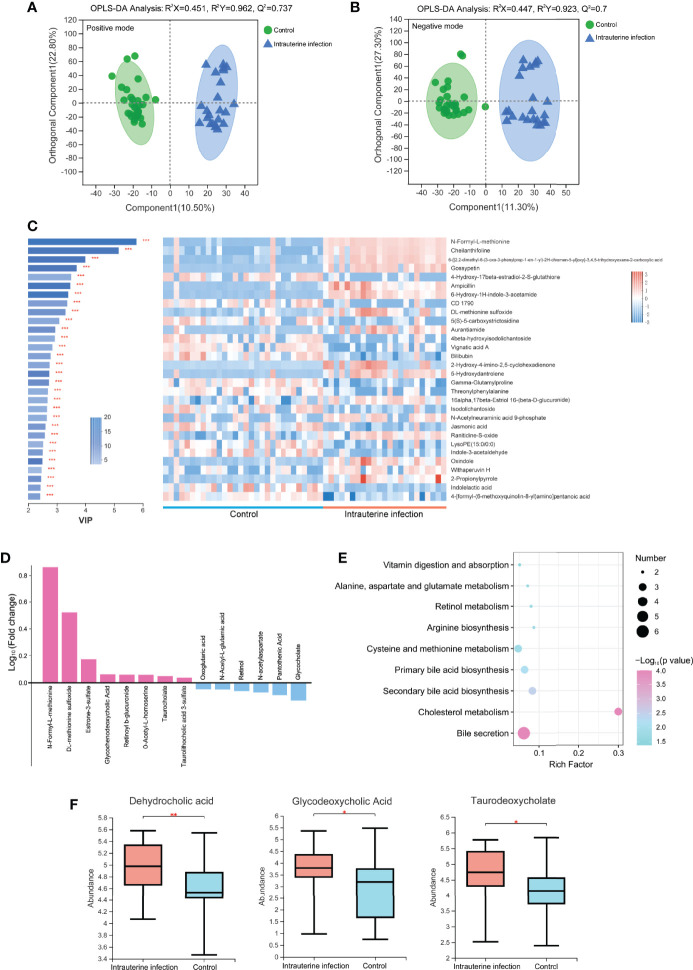
OPLS-DA models of the metabolites between the intrauterine infection group and control group in positive ion mode **(A)** and negative ion mode **(B)**. The variable importance in the projection generated in the OPLS-DA analysis between the intrauterine infection group and control group in positive and negative ion modes **(C)**. **(D)** Log_10_ fold-change of the metabolites involved in vitamin digestion and absorption, retinol metabolism, primary bile acid biosynthesis, secondary bile acid biosynthesis, cholesterol metabolism, bile secretion, cysteine and methionine metabolism, arginine biosynthesis, and the alanine, aspartate, and glutamate metabolism pathways. **(E)** Enriched pathways between the intrauterine infection group and control group in positive and negative ion modes. **(F)** Abundance of dehydrocholic acid, glycodeoxycholic acid, and taurodeoxycholate between the intrauterine infection group and control group. “*” meansP values smaller than 0.05; “**” means P values smaller than 0.01; “***” means P values smaller than 0.001.

A total of 364 metabolites were detected from all samples. OPLS-DA analyses of these metabolites between intrauterine infection group (n = 23 samples) and control group (n = 30 samples) were conducted (**Figures 4A, B**), suggesting that fecal metabolites were significantly different between intrauterine infection group and control group. Permutation testing indicated that the OPLS models were reliable (200 permutations for both) (**Figure S1**).

A total of 304 metabolites were detected in the metabolomic samples of intrauterine infection group (23 samples) and control group (30 samples). The variable importance in the projection (VIP) generated in OPLS-DA revealed the discriminating metabolites (VIP > 1 and *P* < 0.05) of the intrauterine infection group and control group. Among the most discriminant metabolites, N-formyl-L-methionine had the highest VIP value (VIP = 5.7699) (**Figure 4C**). The abundance of N-formyl-L-methionine was significantly higher in the intrauterine infection group than in the control group. This result suggested that this metabolite should be screened in antibiotic-treated neonates of parturients with intrauterine infection. In addition, N-formyl-L-methionine was positively correlated with neonatal neutrophil and WBC (**Figure S2**).

Among the 304 metabolites, the abundance of 129 metabolites was clearly increased in the intrauterine infection group compared with the healthy neonatal group, including retinoyl b-glucuronide, DL-methionine sulfoxide, N-formyl-L-methionine, O-Acetyl-L-homoserine, taurocholate, glycochenodeoxycholic acid, taurolithocholic acid 3-sulfate, and estrone-3-sulfate. In contrast, abundances of 167 metabolites were decreased, including glycocholate, retinol, pantothenic acid, N-acetyl-L-glutamic acid, oxoglutaric acid, and N-acetylaspartate (**Figure 4D**). KEGG pathway analysis showed that these metabolites were involved in nine pathways: vitamin digestion and absorption, retinol metabolism, primary bile acid biosynthesis, secondary bile acid biosynthesis, cholesterol metabolism, bile secretion, cysteine and methionine metabolism, arginine biosynthesis, and the alanine, aspartate and glutamate metabolism pathways (**Figure 4E**). In addition, we found that abundances of dehydrocholic acid, glycodeoxycholic acid, and taurodeoxycholate were significantly higher in the intrauterine infection group than healthy neonatal group (**Figure 4F**).

**Figure 5:**
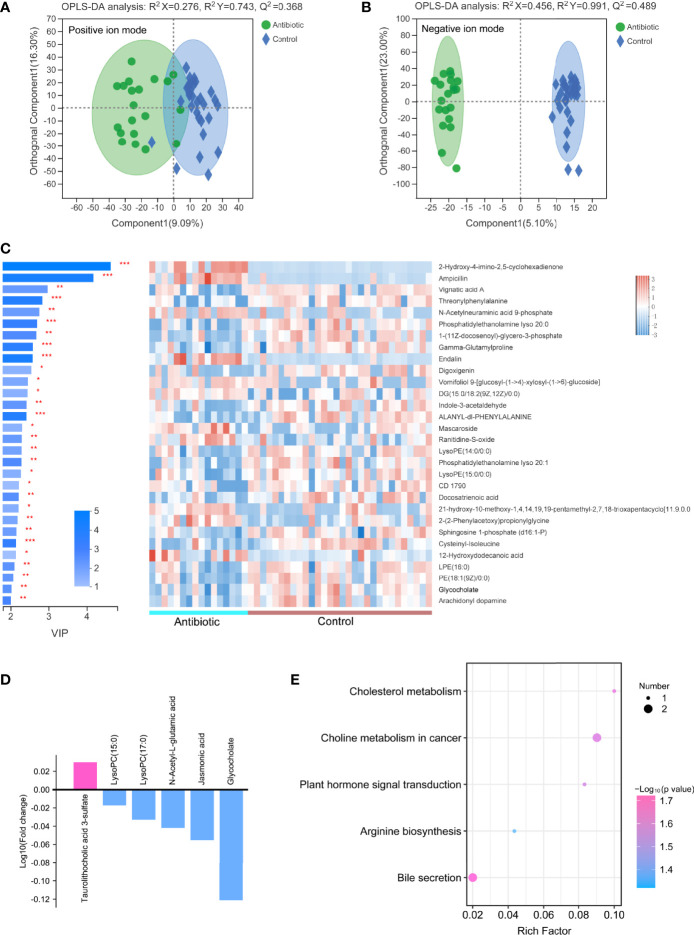
OPLS-DA models of the metabolites between the antibiotic group and control group in positive ion mode **(A)** and negative ion mode **(B)**. The variable importance in the projection generated in the OPLS-DA analysis between the antibiotic group and control group in negative ion mode **(C)**. **(D)** Log_10_ fold-change of the metabolites involved in cholesterol metabolism, plant hormone signal transduction, arginine biosynthesis, bile secretion, and choline metabolism in cancer pathways. **(E)** Enriched pathways between the antibiotic group and control group in negative ion mode. “*” meansP values smaller than 0.05; “**” means P values smaller than 0.01; “***” means P values smaller than 0.001.

The metabolites between the antibiotic and control groups were significantly different in negative ion mode, and were not different in positive ion mode (**Figures 5A, B**). Permutation testing indicated that the OPLS models were reliable (**Figure S3**). 109 metabolites in negative ion mode were detected significantly different between the antibiotic group (16 samples) and the control group, among which the difference trends of 92 metabolites between the two groups were consistent with those between intrauterine infection group and control group, including taurolithocholic acid 3-sulfate, glycocholate, and dehydrocholic acid (**Figure 5C** and **Figure S4**). This result suggests that the differences in these metabolites in the intrauterine infection group might be caused by antibiotic use.

Among the 109 differential metabolites, the abundances of 26 metabolites were increased in the antibiotic group, including taurolithocholic acid 3-sulfate; while 83 metabolites were decreased in the antibiotic group, including jasmonic acid, lysoPC(17:0), lysoPC(15:0), N-acetyl-L-glutamic acid and glycocholate (**Figure 5D**). These six metabolites were involved in five pathways, that were cholesterol metabolism, plant hormone signal transduction, arginine biosynthesis, bile secretion, and choline metabolism in cancer pathways (**Figure 5E**). These results indicate that some changes of metabolic processes (cholesterol metabolism, arginine biosynthesis and bile secretion) might be caused by antibiotic-induced alterations in the gut microbiota.

OPLS-DA analyses of metabolites between intrauterine infection group and antibiotic group suggested that the metabolites were not significantly different between these two groups (**Figure S5**).

### Correlations Between Metabolites and Major Bacterial Genera

**Figure 6:**
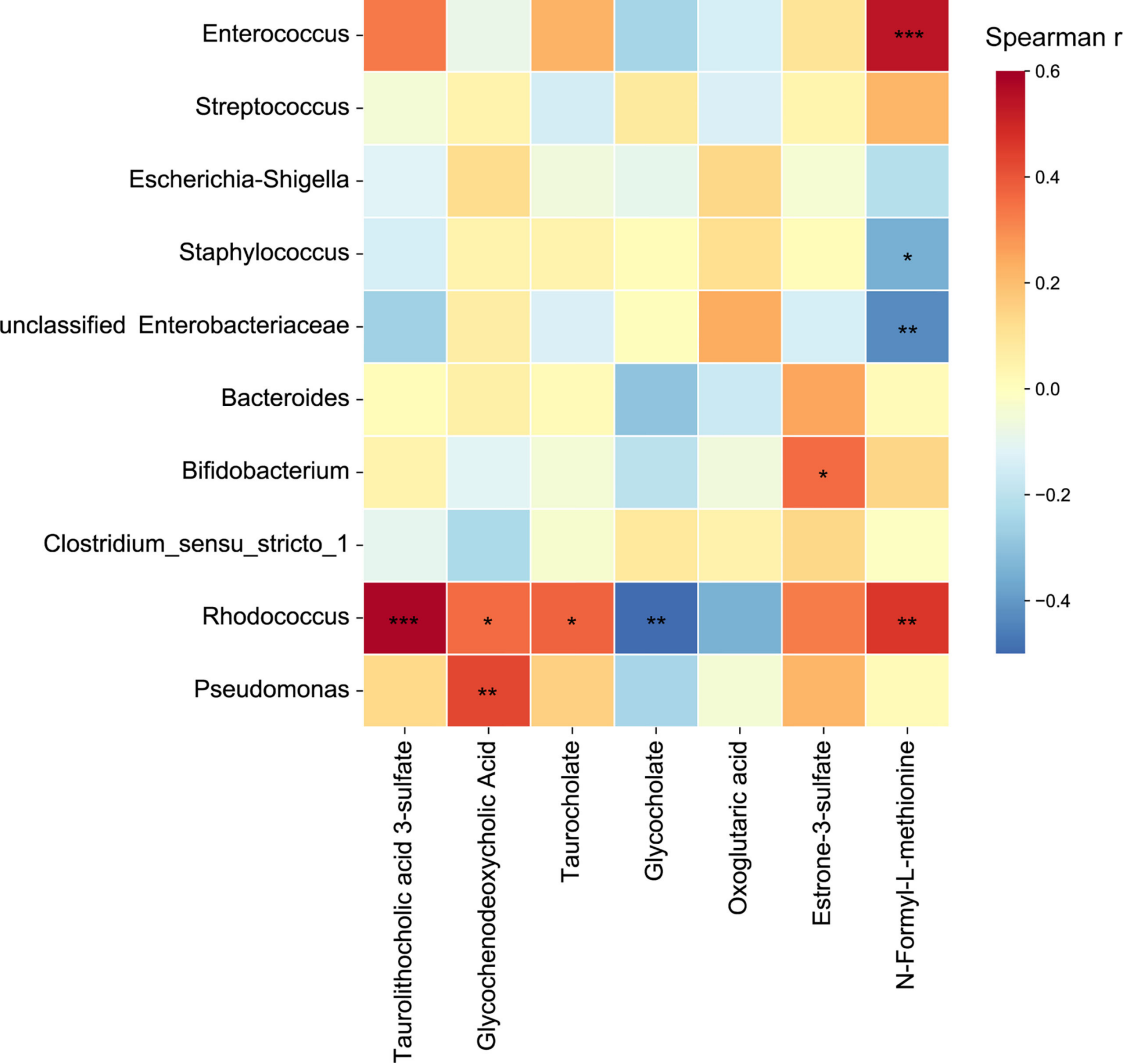
Heatmap of correlation coefficients between the top ten genera and metabolites involved in primary bile acid biosynthesis, secondary bile acid biosynthesis, and bile secretion pathways. Significant correlations are represented by ‘*’ with FDR corrected *P* < 0.05, ‘**’ with FDR corrected *P* < 0.01, and ‘***’ with FDR corrected *P* < 0.001.

Correlations between the abundances of identified metabolites and relative abundances of the top ten genera were calculated. After FDR adjustment, *Enterococcus* and *Rhodococcus* were positively correlated with N-formyl-L-methionine, while *Staphylococcus* and unclassified Enterobacteriaceae were negatively correlated with N-formyl-L-methionine. As for the metabolites involved in the primary bile acid biosynthesis, secondary bile acid biosynthesis, and bile secretion pathways, we found *Rhodococcus* was positively correlated with taurolithocholic acid 3-sulfate, glycochenodeoxycholic acid, and taurocholate, and was negatively correlated with glycocholate (**Figure 6**). This result suggests that *Rhodococcus* plays a role in the bile acid biosynthesis process.

## Discussion

Our results revealed that the gut microbiota of neonates treated with antibiotics born to parturients with intrauterine infection were similar to those born to parturients with non-intrauterine infection, but significantly different from those of healthy control neonates. Antibiotic use may have significant effects, while intrauterine infection alone did not present significant effect on the α-diversity and β-diversity of the gut microbiota, which needs to be further validated in a larger cohort. Compared with healthy neonates, a lower Shannon index (microbial richness and evenness) was found in the intrauterine infection group. In addition, at the phylum level, the abundance of *Firmicutes* increased and the abundance of *Proteobacteria* decreased. Changes in these phyla were found in neonates of parturients with gestational diabetes mellitus (Chen et al., 2021), which might reflect the dysbiosis of gut microbiota (Shin et al., 2015). At the genus level, *Enterococcus* showed the most remarkable discrepancy between the intrauterine infection and control groups, to some extent, which may due to the use of antibiotics. The genus *Enterococcus* includes a group of bacteria that contain antibiotic resistance genes and are resistant to common antibiotics, which may hamper antibiotic therapy in later life stages (Garcia-Solache and Rice, 2019). Our results suggest that dysbiosis of gut microbiota should be considered during neonate antibiotic treatment. Moreover, the relative abundance of *Enterococcus* was positively correlated with neonate neutrophil and WBC counts, which are indicators of infection and inflammation (Huang et al., 2018). This result supports the association between *Enterococcus* and neonatal infection, which has been reported in previous studies (Wang et al., 2014; Subramanya et al., 2019).

Bile acids promote the intestinal absorption of lipids and fat-soluble vitamins, and solubilize cholesterol, and induce bile flow (Gonzalez-Regueiro et al., 2017). In addition, bile acids can regulate the metabolism of lipids, glucose, and energy expenditure (Maruyama et al., 2002; Li and Chiang 2014). In this study, the primary bile acid biosynthesis, secondary bile acid biosynthesis, and bile secretion pathways were enriched in the intrauterine infection group. The abundances of dehydrocholic acid, glycodeoxycholic acid, glycochenodeoxycholic acid, taurodeoxycholate, and taurocholate were higher in the intrauterine infection group. These results indicated that neonates of parturients with intrauterine infection might have a higher risk of developing metabolic disorders later in life. What’s more, high concentration of bile acids has cytotoxicity and may lead to inflammation in intestinal tissue (Poupon et al., 2000). Moreover, bile acids have antibacterial activity (Guzior and Quinn, 2021). Thus, bile acids might disrupt the gut microbiota of neonates, although they can help innate intestinal immune defense (Guzior and Quinn, 2021). Taken together, these results indicate the possibility of an adverse impact caused by abnormalities in intestinal bile acids and bile salts in neonates of parturients with intrauterine infection. On the other hand, we found that *Rhodococcus* was positively correlated with taurolithocholic acid 3-sulfate, glycochenodeoxycholic acid and taurocholate, and was negatively correlated with glycocholate. Previous studies have shown that some *Rhodococcus* strains could degrade bile acids (Mohn et al., 2012). However, this could not fully explain the positive and negative correlations between *Rhodococcus* and bile acids and bile salts. Further mechanism research should be performed to investigate how *Rhodococcus* spp. are involved in gut bile acid metabolic processes.

N-formyl-L-methionine is capable of chemotactic neutrophils and is involved in the inflammatory response (Ackerman and Douglas, 1979). In this study, N-formyl-L-methionine was positively correlated with neonatal neutrophil and WBC levels, suggesting that it is involved in the inflammatory response. On the other hand, N-formyl-L-methionine is a precursor of L-methionine, which is an essential amino acid for protein synthesis. It’s interesting that the abundance of N-formyl-L-methionine was higher in the intrauterine infection group. In addition, we found that N-formyl-L-methionine was positively correlated with *Enterococcus* and *Rhodococcus*, and was negatively correlated with *Staphylococcus* and unclassified Enterobacteriaceae. Currently, little is known about the relationship between N-formyl-L-methionine and the gut microbiota and the mechanism by which this metabolite interacts with the gut bacteria is unknown. Future work should be conducted to investigate the role of N-formyl-L-methionine in the gut environment of neonates. Together, our results indicate that N-formyl-L-methionine in neonates treated with antibiotics should be screened to ensure their well-being.

One limitation of this study is that our results may only reflect neonatal gut microbiota and gut metabolites near the treatment time, and may not indicate the long-term effects of the treatment. Longitudinal samples should be involved to investigate the long-term effects in further studies. Another limitation is that the effects on gut microbiota may be specific for beta-lactam antibiotics, because the neonates in the intrauterine infection group and antibiotic group were only treated with beta-lactam. It’s necessary to investigate the effects of other antibiotics in more studies.

In conclusion, our results provide a preliminary description of the gut microbiota and gut metabolites in antibiotic-treated neonates of parturients with intrauterine infection. Dysbiosis of gut microbiota in neonates should be considered during antibiotic treatment. Although the metabolomics data need to be further validated, our results show that the metabolite profiles of the intrauterine infection group were significantly different from those of the healthy group, and that several KEGG metabolic pathways related to bile acid were altered.

## Data Availability Statement

The datasets presented in this study can be found in online repositories. The names of the repository/repositories and accession number(s) can be found in the article/**Supplementary Material**.

## Ethics Statement

The studies involving human participants were reviewed and approved by Medical Ethics Committee of the Affiliated Shenzhen Maternity & Child Healthcare Hospital. Written informed consent to participate in this study was provided by the participants’ legal guardian/next of kin.

## Author Contributions

HL and YC contributed to the study design. HL, XC, and HX contributed to the diagnosis and sample collection. XC and LF contributed to the data analysis. QJ, CY, ZW, and YC contributed to manuscript writing and revisions and approved the final manuscript. All authors contributed to the article and approved the submitted version.

## Funding

This study was supported by the Guangzhou Science and Technology Project (202102020342), Guangdong Basic and Applied Basic Research Foundation (2020B1515120034), Shenzhen Science and Technology Program (JCYJ20180306173125699), and Shenzhen Fund for Guangdong Provincial High level Clinical Key Specialties (No.SZGSP009).

## Conflict of Interest

The authors declare that the research was conducted in the absence of any commercial or financial relationships that could be construed as a potential conflict of interest.

## Publisher’s Note

All claims expressed in this article are solely those of the authors and do not necessarily represent those of their affiliated organizations, or those of the publisher, the editors and the reviewers. Any product that may be evaluated in this article, or claim that may be made by its manufacturer, is not guaranteed or endorsed by the publisher.
