# Supplementary material for: Gut Microbiota and Metabolome Description of Antibiotic-Treated Neonates From Parturients With Intrauterine Infection
Source: Front Cell Infect Microbiol. 2022 Mar 18;12:817832. doi: 10.3389/fcimb.2022.817832 (PMC8974630; doi:10.3389/fcimb.2022.817832)
Supplement: Supplementary file 1 [file DataSheet_1.docx]

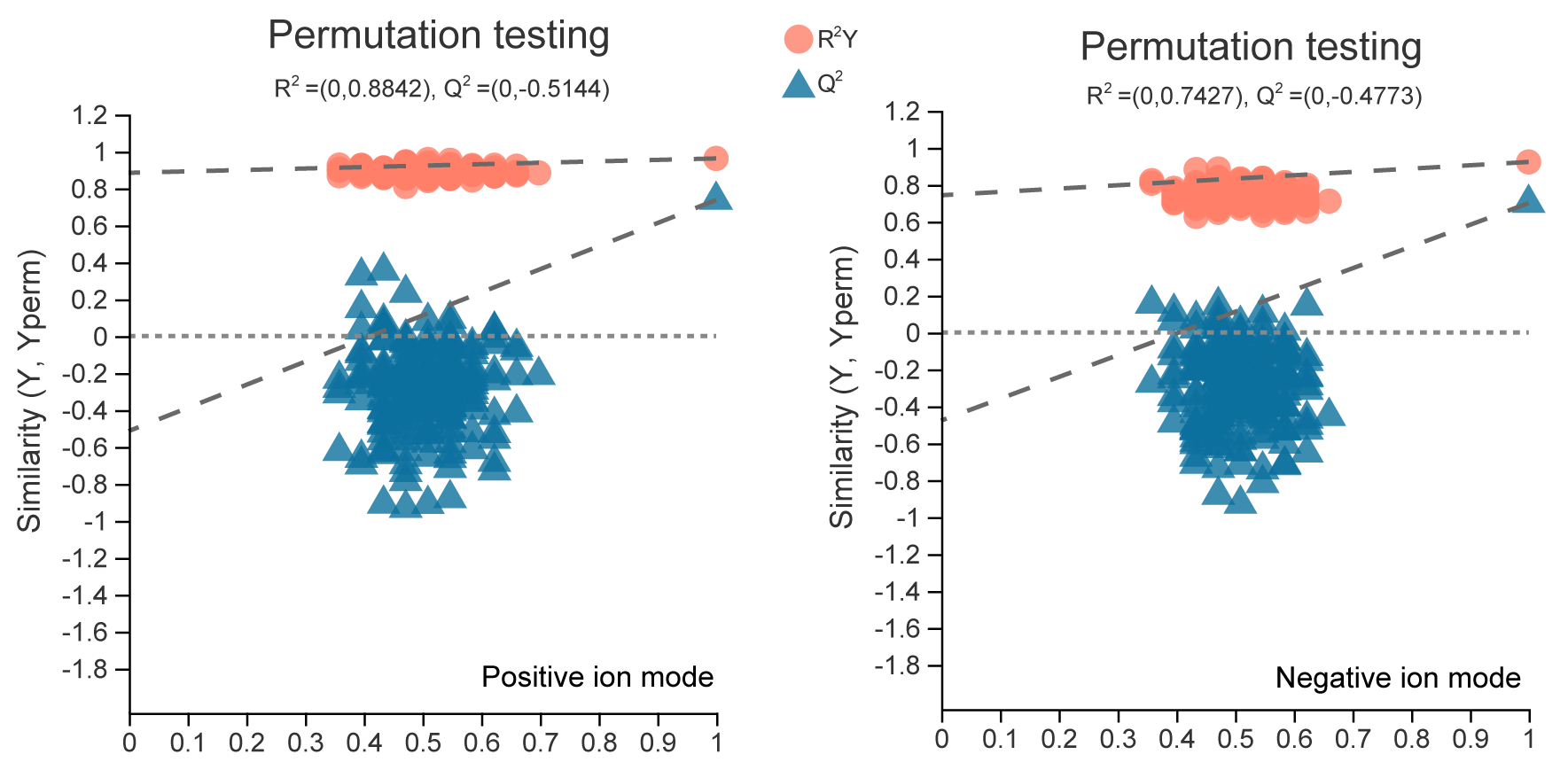


**Supplementary Figure 1:** Permutation tests of the metabolites between the intrauterine infection group and control group in positive and negative ion modes.


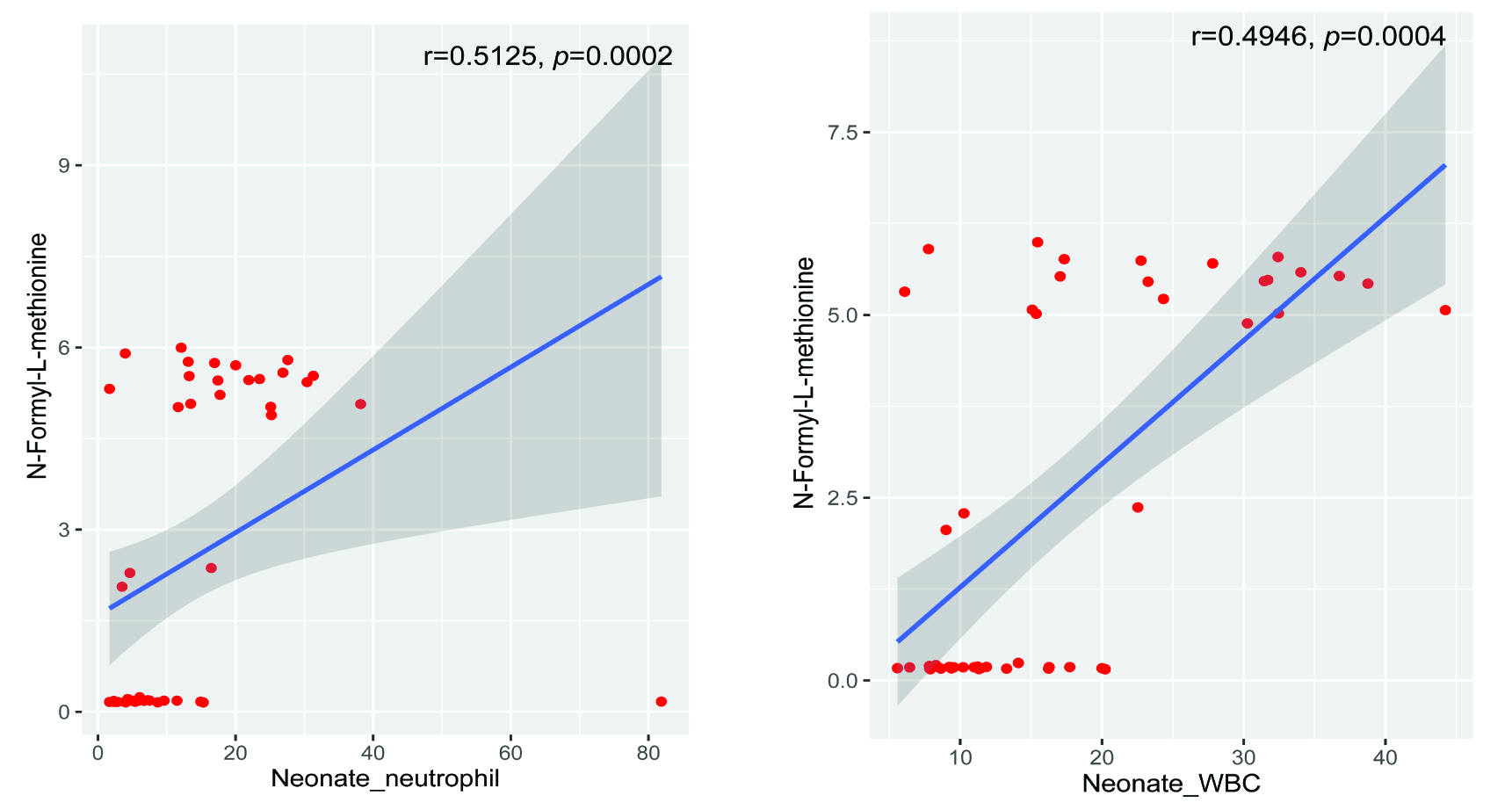


**Supplementary Figure 2:** Spearman’s correlations between N-formyl-L-methionine and neonatal neutrophil and between N-formyl-L-methionine and neonatal white blood cell counts.


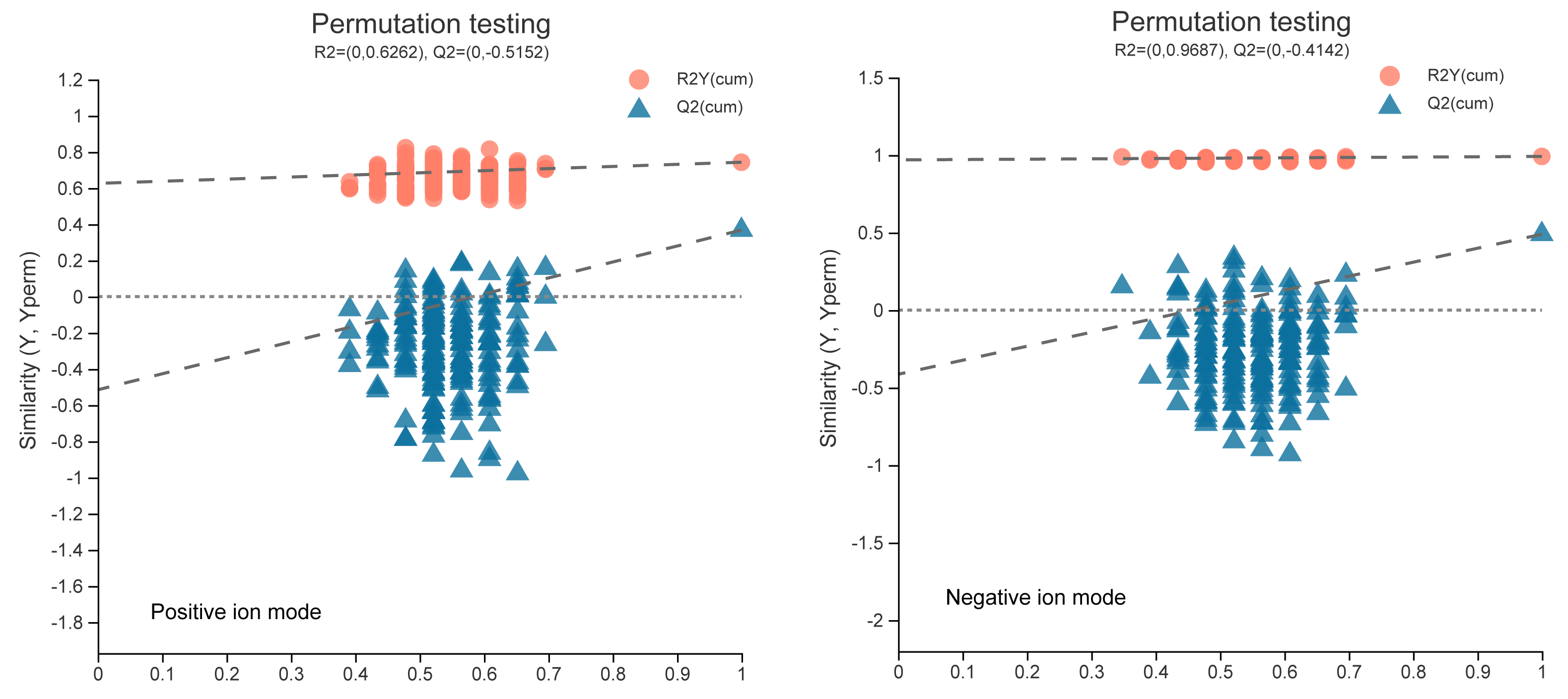


**Supplementary Figure 3:** Permutation tests of the metabolites between the antibiotic group and control group in positive and negative ion modes.


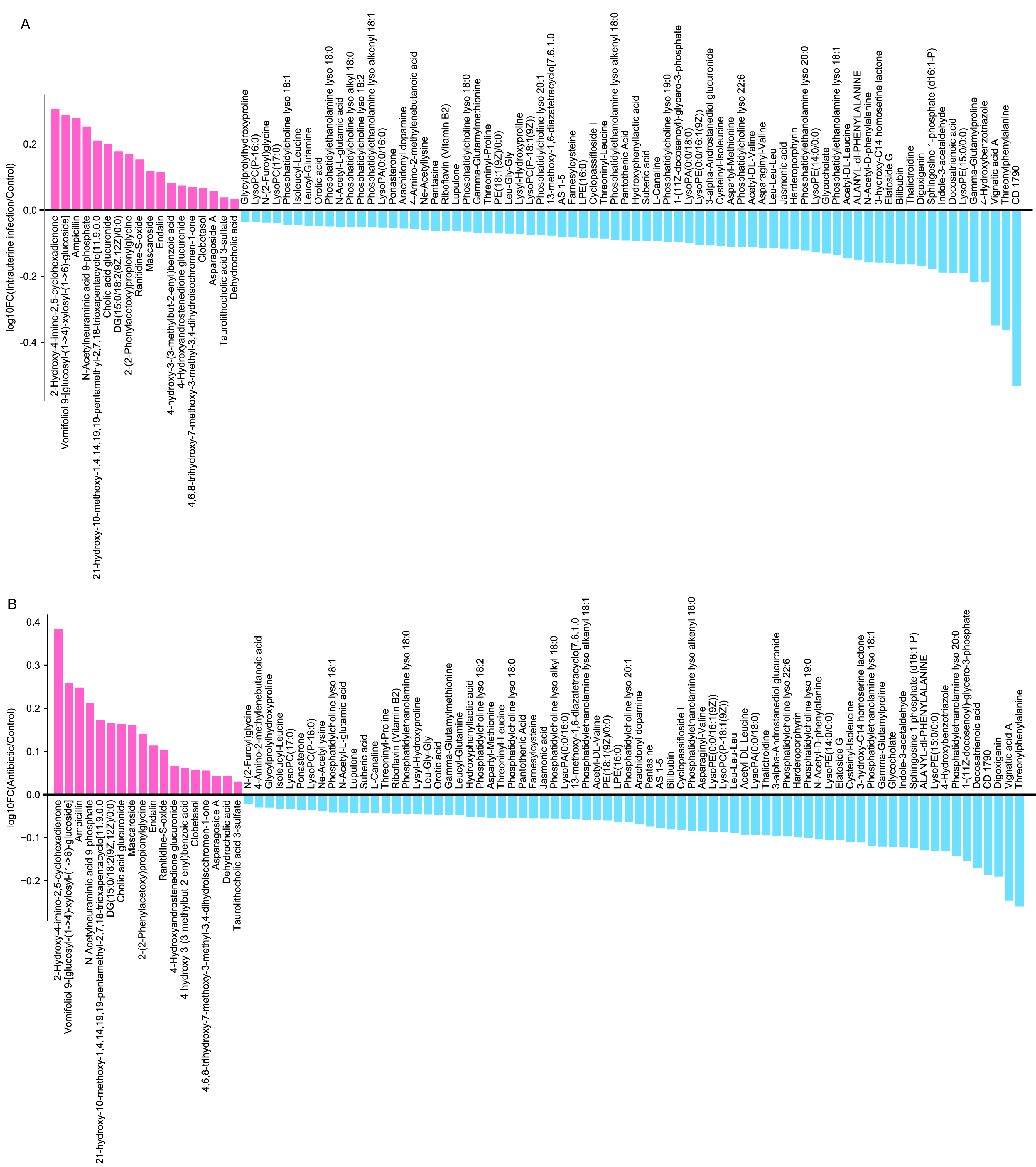


**Supplementary Figure 4:** Different trends of 92 metabolites between the intrauterine infection group and control group (**A**) were consistent with those between the antibiotic group and control group (**B**).


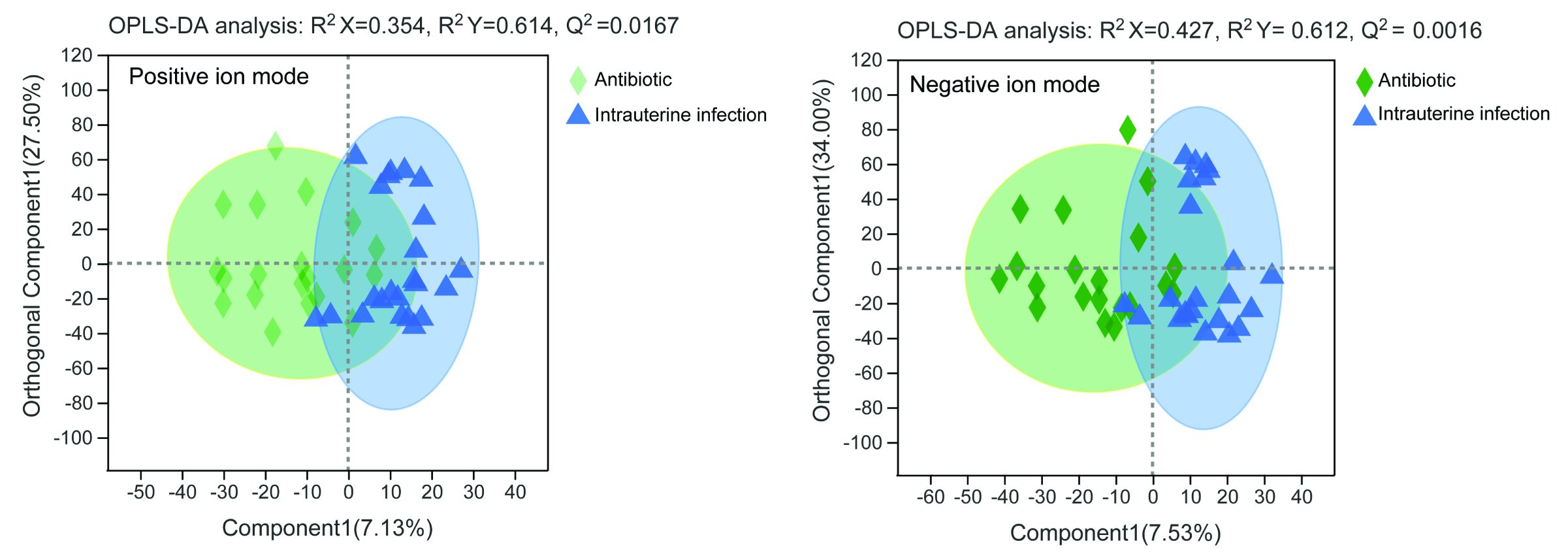


**Supplementary Figure 5:** OPLS-DA models of the metabolites between the infection group and antibiotic group in positive ion mode **(A)** and negative ion mode **(B)**.
