# Supplementary material for: Gut Microbiota and Metabolome Description of Antibiotic-Treated Neonates From Parturients With Intrauterine Infection
Source: Front Cell Infect Microbiol. 2022 Mar 18;12:817832. doi: 10.3389/fcimb.2022.817832 (PMC8974630; doi:10.3389/fcimb.2022.817832)
Supplement: Supplementary file 2 [file DataSheet_2.docx]

**SUPPLEMENTAL METHODS：**

**Detailed Participant Information.**

Intrauterine infection was defined according to the Chinese Obstetrice and Gynecology based on a maternal meeting of three of the seven following criteria: (1) maternal temperature ≥ 37.5 ℃, (2) maternal heart rate > 100 per minute, (3) fetal heart rate > 160 per minute, (4) foul-smelling amniotic fluid, (5) uterine tenderness, (6) blood WBC > 15×10^9^/L, (7) C-reactive protein > 8 mg/L. Parturients in the control group were healthy parturients. No subject received antibiotics during their late pregnancy. Women with diabetes, metabolic diseases, antibiotics usage within 3 months, alcohol or substance abuse, and chronic diseases requiring medication were excluded. Neonates with deformity, multiple births, diarrhea, and constipation were excluded.

In this study, intrauterine infection group (n = 23) were neonates treated with beta-lactam antibiotics from parturients with intrauterine infection. All neonates of the intrauterine infection group received beta-lactam antibiotics after birth. Fecal samples of all neonates in the intrauterine infection group were collected during the course of their antibiotic treatment. The antibiotic group (n = 16) were neonates from non-intrauterine infection parturients with indication for antibiotic use, such as amniotic fluid aspiration and meconium aspiration. Neonates of the antibiotic group were treated with beta-lactam antibiotics. Fecal samples of twelve neonates in the antibiotic group were collected during the course of their antibiotic treatment, and samples of four neonates were collected after the treatment course (less than 48 hours). The control group (n = 30) were neonates from health parturients without indication for antibiotic use.

Fecal samples and blood samples from all neonates were collected on the same day at the first week after birth. Each fecal sample was divided into two parts (one for gene sequencing and one for untargeted metabolomics) and stored at -80 ℃ within one hour. Laboratory measurements of blood neutrophils and WBC counts were conducted at the clinical lab of Shenzhen Maternity and Child Healthcare Hospital.

**DNA extraction and 16S rRNA gene sequencing**.

DNA extraction was performed using a Mag-Bind Soil DNA Kit (Omega Bio-Tek, USA). The V4 region of the bacterial 16S rRNA gene was amplified using the barcoded primers 514F (5’-GTGCCAGCMGCCGCGGTAA-3’) and 805R (5’-GGACTACHVGGGTWTCTAAT-3’) (Walters et al., 2016). Among the PCR products of 23 fecal samples from intrauterine infection group, 18 PCR products met the sequencing requirement. Among the PCR products of 16 fecal samples from antibiotic group, 12 met the sequencing requirement. All PCR products of the 30 control group samples met the sequencing requirement. These 60 PCR products were pooled in equimolar and paired-end sequenced on an Illumina MiSeq PE300 platform (Illumina, San Diego, USA) according to the standard protocols by Majorbio Bio-Pharm Technology Co. Ltd. (Shanghai, China). The raw reads were deposited into the European Nucleotide Archive database (Accession Number: PRJEB48156).

**Processing of 16S rRNA gene sequencing data**.

The processing of sequencing data were similar to that previously described (Zheng et al., 2020). The raw sequencing data were demultiplexed, quality-filtered by fastp (version 0.20.0) (Chen et al., 2018) and merged by Flash (version 1.2.11) (Magoc et al., 2011) with the following criteria: (i) the 300 bp reads were truncated at any site receiving an average quality score of < 20 over a 50 bp sliding window, and the truncated reads shorter than 50 bp were omitted out, reads containing ambiguous characters were also omitted; (ii) only overlapping sequences longer than 10 bp were assembled according to their overlapped sequence. The maximum mismatch ratio of overlap region is 0.2. Reads that could not be assembled were omitted; (iii) samples were distinguished according to the barcode and primers, and the sequence direction was adjusted, exact barcode matching, 2 nucleotide mismatch in primer matching.

OTUs with 97% similarity cutoff were clustered using Uparse (version 7.0.1090) (Edgar, 2013). The taxonomy of OTU was analyzed by RDP Classifier (version 2.11) (Edgar, 2013).

**Gut microbiota analysis**.

Each sample was normalized to 53201 sequences for the subsequent analyses. QIIME (version 1.9.1) was subsequently used to analyse the sequences (Caporaso et al., 2010). Adonis tests were used to analyze Bray–Curtis and Euclidean distances on OTU level in three groups. Kruskal-Wallis tests were used to analyze the relative abundances of phyla and genera among three groups. Mann-Whitney U tests were used to analyze relative abundances of genera between two groups. Spearman’s rank correlation tests were used to examine the correlations between genera and neonatal neutrophil and correlations between genera and neonatal WBC counts.

**Untargeted metabolomics by LC-MS**.

Sample preparation and LC-MS were similar to that previously described (Ding et al., 2020; Zhao et al., 2020), and were conducted by Majorbio Bio-Pharm Technology Co. Ltd. (Shanghai, China). Fecal samples (50 mg for each) was dissolved in 400 μL of extracting solution (1:1 mixture of acetonitrile and methanol). Then the samples were vortexed for 30 s and metabolites were extracted by sonication in an ice water bath for 30 min. Then the samples were incubated at −20 °C for 30 min and were centrifuged (13000 g) for 15 min. The supernates of each sample were used for LC-MS. LC-MS analysis was performed on a ultra performance liquid chromatography system (Waters, Milford, USA) coupled with a time of flight mass spectrometer (Triple TOF 5600, AB SCIEX, USA). The quality control sample was a mixture of all sample extraction aliquots.

**Data analysis of metabolites**.

The raw data in LC-MS (23 samples in intrauterine infection group, 16 samples in antibiotic group, and 30 samples in control group) were imported into Progenesis QI software (Waters, Milford, USA). The peak intensity was normalized. The peaks with an RSD of higher than 30% in the QC samples were excluded. Logarithmic transformation (Log_10_) was conducted and the data matrix for subsequent analysis was obtained. Metabolites identification and pathway analysis were conducted on Metaboanalyst (http://www.metaboanalyst.ca) and KEGG database (version 94.2).

Orthogonal partial least squares-discriminant analysis (OPLS-DA) was used to distinguish the alterations between two groups. Metabolites with variable importance in the projection (VIP) > 1 and p < 0.05 were identified as significantly changed metabolites between two groups. No clustering algorithm was used. Metabolic pathways were annotated by KEGG database to obtain the pathways involved in differential metabolites. The Python software package scipy (version 1.0.0) was used to conduct pathway enrichment analysis.

Unpaired Wilcox’s tests were used to compare the abundances of metabolites in two groups. Spearman’s rank correlation tests were used to analyze the correlations between metabolites and neonatal neutrophil and correlations between metabolites and neonatal WBC counts.

**References:**

Caporaso, J. G., Kuczynski, J., Stombaugh, J., Bittinger, K., Bushman, F. D., and Costello, E. K., et al. (2010). QIIME allows analysis of high-throughput community sequencing data. *Nat. Methods*. 7(5), 335-336. doi: 10.1038/nmeth.f.303

Chen, S., Zhou, Y., Chen, Y., Gu, J. (2018). Fastp: An ultra-fast all-in-one FASTQ preprocessor. *Bioinformatics*. 34(17), i884-i890. doi: 10.1093/bioinformatics/bty560

Ding, J., Huang, Y., Liu, S., Zhang, S., Zou, H., and Wang, Z., et al. (2020). Toxicological effects of nano- and micro-polystyrene plastics on red tilapia: Are larger plastic particles more harmless? *J. Hazard. Mater.* 396, 122693. doi: 10.1016/j.jhazmat.2020.122693

Edgar, R. C. (2013). UPARSE: Highly accurate OTU sequences from microbial amplicon reads. *Nat. Methods*. 10(10), 996-998. doi: 10.1038/nmeth.2604

Magoc, T., Salzberg, S. L. (2011). FLASH: Fast length adjustment of short reads to improve genome assemblies. *Bioinformatics*. 27(21), 2957-2963. doi: 10.1093/bioinformatics/btr507

Walters, W., Hyde, E. R., Berg-Lyons, D., Ackermann, G., Humphrey, G., and Parada, A., et al. (2016). Improved bacterial 16S rRNA gene (V4 and v4-5) and fungal internal transcribed spacer marker gene primers for microbial community surveys. *mSystems*. 1(1). doi: 10.1128/mSystems.00009-15

Wang, Q., Garrity, G. M., Tiedje, J. M., Cole, J. R. (2007). Naive Bayesian classifier for rapid assignment of rRNA sequences into the new bacterial taxonomy. *Appl Environ Microbiol*. 73(16), 5261-5267. doi: 10.1128/AEM.00062-07

Zhao, H. J., Xu, J. K., Yan, Z. H., Ren, H. Q., Zhang, Y. (2020). Microplastics enhance the developmental toxicity of synthetic phenolic antioxidants by disturbing the thyroid function and metabolism in developing zebrafish. *Environ. Int.* 140, 105750. doi: 10.1016/j.envint.2020.105750

Zheng, P., Yang, J., Li, Y., Wu, J., Liang, W., and Yin, B., et al. (2020). Gut Microbial Signatures Can Discriminate Unipolar from Bipolar Depression. *Adv Sci (Weinh)*. 7(7), 1902862. doi: 10.1002/advs.201902862
